# Study Protocol for the Development and Preliminary Efficacy Assessment of *AdoptMindful2Care@Web*: A Web-Based Mindful Parenting Postadoption Intervention

**DOI:** 10.3390/healthcare12212100

**Published:** 2024-10-22

**Authors:** Ana Luz Chorão, Maria Cristina Canavarro, Raquel Pires

**Affiliations:** Center for Research in Neuropsychology and Cognitive-Behavioral Intervention (CINEICC), Faculty of Psychology and Educational Sciences, University of Coimbra, 3000-115 Coimbra, Portugal; ana.chorao@student.fpce.uc.pt (A.L.C.); mccanavarro@fpce.uc.pt (M.C.C.)

**Keywords:** adoptive parents, content and technological feature development, e-health technology, mental health promotion, efficacy assessment, mindful parenting, user-centered approach

## Abstract

Developing postadoption interventions to prevent parenting stress and promote parents’ well-being is urgent. Mindful parenting-based interventions show promise in achieving these goals and are well received by adoptive parents (APs). However, face-to-face interventions face significant barriers. E-health tools offer a solution by improving accessibility and flexibility and reducing long-term costs. Our main aim is to develop and assess the preliminary efficacy of a web-based mindful parenting postadoption intervention, *AdoptMindful2Care@Web*, by using a user-centered approach to ensure its effectiveness and cost efficiency. First, two quantitative surveys will be conducted to assess the needs and preferences of APs and gather input from adoption professionals. Then, a prototype version of the intervention will be created and tested for usability with the APs via the Think-Aloud method. Finally, a pilot version will undergo a two-arm pilot randomized controlled trial to assess its feasibility, acceptability, and preliminary efficacy via self-report questionnaires. By developing *AdoptMindful2Care@Web* employing a user-centered approach, we hope to provide an effective intervention that is adjusted to the reality of its users and the surrounding context. In doing so, we will be able to promote AP access to specialized mental health care efficiently.

## 1. Introduction

Adoptive parents seem to be particularly vulnerable to depression, anxiety, stress, and difficulties in being emphatic, sensitive, and efficient in managing children’s behavior [1]. Such parents are obliged to address adoption-related issues (e.g., experiences with infertility, increased likelihood of parenting children with preexisting behavioral/emotional difficulties, and stigma attached to adoption [2,3,4]), which can place them in a more vulnerable position with respect to these mental health issues than nonadoptive parents. This is a particularly concerning reality, since it is thoroughly reported in the literature that these challenging circumstances can compromise children’s development and adoption stability [5]. However, in Portugal, there are no structured governmental postadoption services yet available, although a legal framework for the provision of postadoption support was set out in 2015 [6]. Similarly, there are no evidence-based psychological interventions specifically designed to promote adoptive parents’ well-being and positive parenting available in our country.

Worldwide, postadoption therapeutic services, when available, are usually focused on children’s difficulties, parents’ ability to manage children’s behavior, and parent-child relationship quality within an attachment framework [7,8,9]. These interventions are indispensable, but they are not designed to incorporate parents’ needs in coping with the emotional challenges of parenthood or in achieving greater nonjudgmental acceptance of and compassion for the self and the child, or self-regulation of immediate emotional states in favor of long-term goals that sustain the parent-child relationship [10]. These factors may be crucial targets for reducing parenting stress, promoting positive parenting, and thus improving families’ well-being [11,12].

Mindful parenting interventions (MPIs) are focused on the targets mentioned above [13] and are highly acceptable among adoptive parents, as well as promising in promoting both adoptive parents’ and children’s well-being [14,15,16]. However, insurmountable barriers arise when planning the face-to-face provision of such interventions in Portugal. This issue is related to the fact that in Portugal, 18 social security district centers (SSDCs) ensure postadoption support in different geographic reference areas; each one has coordinated but independent organizational structures and procedures, serves heterogeneous populations, and faces specific time, material, and human constraints in providing postadoption support. Regarding therapeutic interventions specifically, several shared constraints can be noted: (1) severe discrepancies in the number of adoptions carried out in each SSDC, high geographic dispersion, and different adoption timings among adoptive families, for example, constrain establishing face-to-face therapeutic group formats, and (2) severe time, material, and human limitations and reduced expertise in therapeutic care by the adoption teams constrain the individual face-to-face application of MPIs [17]. These circumstances call for alternative delivery formats.

E-mental health (e-mhealth) tools (i.e., the use of information technologies to deliver mental health services at a distance [18])—namely, web-based interventions (i.e., self-guided treatments delivered via websites and designed to educate and/or cause therapeutic change [19])—are an innovative form of intervention and seem to be a promising option to overcome most of the noted barriers, particularly given their many benefits: (1) improved access to care, since health care can become available regardless of time and place, contributing to health care equity and removing thresholds such as stigmatization; (2) empowerment, giving people the opportunity to take greater control of their own health care by enabling them to choose when and where they want to access it; (3) innovation, since these technologies open up a wide range of possibilities for health care and can provide the groundwork for sustainable changes; and (4) quality of care, owing to highly efficient and innovative systems and to effective interventions that lower costs and increase safety by reducing human errors [20]. These tools are already being used with adoptive parents in other countries [21,22]; however, none of them were specifically designed to help adoptive parents deal with the emotional challenges of parenthood or adopt mindful parenting practices; additionally, they are not prepared to be used in environments other than those for which they were designed—which makes their replication in the Portuguese context unfeasible. However, these interventions may offer valuable insights into features that should be considered in the development of a new intervention of this nature [23,24].

The development of e-mhealth tools is a complex process that requires a user-centered approach to incorporate the requisite people–context–technology interrelationships, i.e., several steps must be followed during content and technological feature development with the active participation of prospective users and other key stakeholders [20]. According to the guidelines of the Management Centre for e-Health Research and Disease (CeHRes) Roadmap [25,26], a reliable and useful product is directly dependent on the contextual inquiry, value specification, and design phases, since they enable the understanding and incorporation of the physical–social–cultural environment and the needs and preferences of prospective users, funders, and policy-makers that the product should serve through its content and technological features. This work is even more important when few studies have been performed with prospective users, as is the case for Portuguese adoptive parents [20,27].

By developing and preliminarily testing the efficacy of a web-based mindful parenting postadoption intervention, *Adoptindful2Care@Web*, employing a user-centered approach, our project intends to respond efficiently to the urgent need to improve adoptive parents’ access to specialized mental health care [28]. To better ensure *Adoptindful2Care@Web* suitability, usefulness, efficacy, and cost-effectiveness, adoptive parents and adoption professionals will be involved throughout the entire development (i.e., contextual inquiry, value specification, and design) and preliminary evaluation process. Here, we describe the study protocol for the content and technological feature development and the preliminary efficacy assessment of *AdoptMindful2Care@Web*.

## 2. Materials and Methods

### 2.1. Design

*AdoptMindful2Care@Web* will be developed following the CeHRes Roadmap and by adapting the structure of its standard version (*AdoptMindful2Care*; [29]) according to the results of the current protocol.

*AdoptMindful2Care* is an eight-session, face-to-face group intervention in which adoptive parents learn to apply mindfulness and self-compassion skills to themselves and to their parenting experience. This intervention was built on the basis of the Mindful Parenting Program manual [13]. The following topics are covered: (1) automatic pilot parenting, (2) beginner’s mind parenting, (3) reconnecting with our body as a parent, (4) responding versus reacting to parenting stress, (5) parenting patterns and schemas, (6) conflict and parenting, (7) love and limits, and (8) a mindful path through parenting.

In *AdoptMindful2Care@Web*, we intend to keep the same content (each topic will correspond to a module), adapting its presentation format (e.g., through videos recorded by psychologists, audios, interactive texts, and interactive exercises) according to the results obtained in the exploratory studies we will conduct at the beginning of the project (Studies 1 and 2).

**Figure A1:**
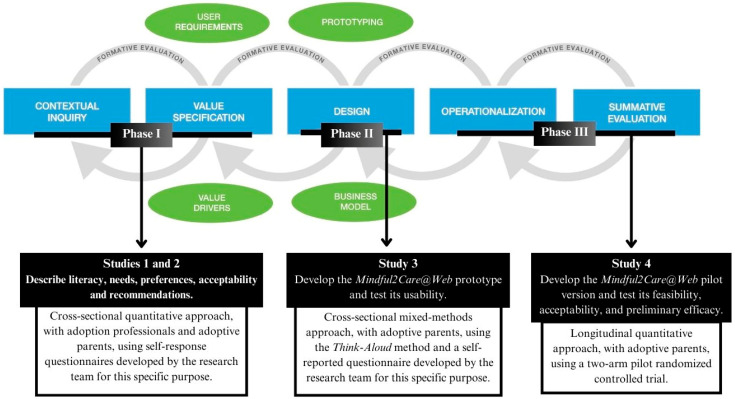
Our study protocol integrated into the CeHRes Roadmap.

The CeHRes Roadmap [11] provides an evidence-based development approach for e-health interventions. It states that the development of an e-health intervention must incorporate the contributions of prospective users and other stakeholders throughout the entire creation and pre-testing process, which includes the contextual inquiry, value specification, and design phases. By creating a good fit among the human, technological, and contextual factors, this holistic approach can increase the degree to which an intervention reaches its goals. The development of *AdoptMindful2Care@Web* will be guided by a user-centered approach, which will rely on the active participation of adoptive parents—as its future users—and professionals working in adoption teams—as expected mediators of access to the intervention—through the entire process (Figure A1).

To achieve this goal, the present study protocol will be divided into three phases:

- Phase I, which will be dedicated to contextual inquiry and value specification tasks. In turn, this phase is divided into two distinct but complementary studies:
  o Study 1, performed with adoption professionals, will use a survey developed by the research team for this purpose and is intended to explore the recommendations of such professionals regarding the content, format, and dissemination features required for AdoptMindful2Care@Web to meet (1) the needs expressed by adoptive parents when asking for professional support, (2) the professionals’ own needs when providing support for adoptive parents, and (3) the adoption system’s ethical and institutional requirements for the integration of AdoptMindful2Care@Web in the organizational structure and working routine of adoption services.
  o Study 2, performed with adoptive parents as prospective users of AdoptMindful2Care@Web, will use a survey developed by the research team for this purpose to (1) characterize the literacy of adoptive parents regarding e-mhealth tools, (2) identify the mental health and parenting needs that such parents expect to be addressed by AdoptMindful2Care@Web, and (3) analyze the preferences and acceptance (perceived utility and availability for use) of such parents regarding AdoptMindful2Care@Web’s content, format, and dissemination features.
- Phase II, which will be dedicated to the design task. In this phase, the prototype of *AdoptMindful2Care@Web* will be developed on the basis of the information gained in Phase 1. The intervention’s content will be developed by the research team and systematically and interactively reviewed by researchers/clinicians in the adoption field. Technological features will be developed by a specialized IT company according to the guidance of the research team. This process will be monitored by LAICOS–Behavioral Change, a company specialized in behavioral science. In this phase, we will also perform Study 3, which is intended to test the usability of the prototype version of *AdoptMindful2Care@Web* with adoptive parents, using the Think-Aloud Protocol and a survey developed by the research team for this purpose.
- Phase III, which will be dedicated to operationalization and preliminary summative evaluation. The pilot version of *AdoptMindful2Care@Web* will be developed on the basis of the results of the previous phase. Then, we will perform Study 4, which is intended to assess the feasibility, acceptability, and preliminary efficacy of the intervention (i.e., improvements in parent and child outcomes at postintervention and/or follow-up) through a two-arm pilot randomized controlled trial. The results of this study will inform the necessary changes to be incorporated into *AdoptMindful2Care@Web* to establish its final version.

**Figure 1:**
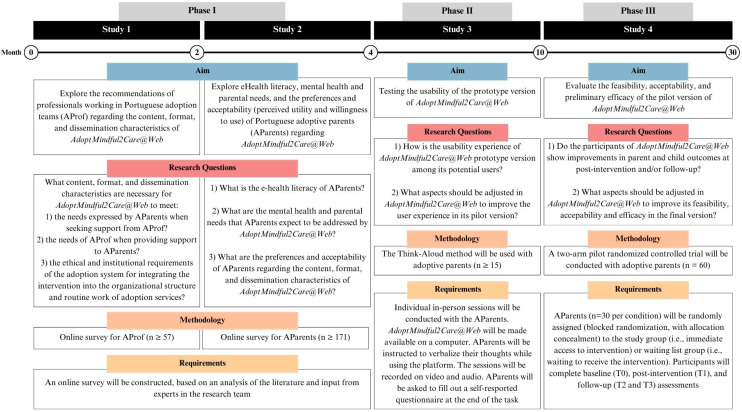
An overview of the studies included in the protocol.

See Figure 1 for a detailed overview of the study protocol.

### 2.2. Participants

The participants in Study 1 will be professionals who will have worked in Portuguese adoption services for at least 2 years.

In Studies 2, 3, and 4, the participants will be adoptive parents. To be eligible, parents will have to have (a) at least one adoptive child aged under 18 years; (b) no other adoption process in progress; (c) reading and writing abilities in the Portuguese language; and (d) regular access to the internet and electronic devices. In Studies 3 and 4, parents will be excluded if they will have been diagnosed with a serious mental health condition (e.g., schizophrenia).

### 2.3. Materials

The instruments used and the respective outcomes measured are presented in Table 1.

### 2.4. Procedure

This three-phase, multimethod study protocol is supported by the Portuguese Foundation for Science and Technology through a doctoral grant. It is part of a larger project hosted in the Faculty of Psychology and Education Sciences of the University of Coimbra and supported by the Calouste Gulbenkian Foundation. It will be carried out with the collaboration of scientific and community institutions active in the fields of adoption and parenting and of all the Portuguese adoption agencies. The data are planned to be collected up to May 2026.

For Studies 1, 2, and 4, self-report surveys provided by a secure online survey tool of the host institution (LimeSurvey^®^) will be used. Data will be stored on this platform in accordance with all relevant confidentiality and privacy rules. For Study 3, the Think-Aloud Protocol method will be used.

Participants will be recruited through adoption agencies. Potential participants will be selected by these agencies, which will subsequently send an e-mail to them (written by the research team).

In Studies 1 and 2, the e-mail will contain summary information about the objectives of the study and the researchers’ contacts, as well as the link to the online survey. The survey is estimated to take an average of 30 min to complete. The participants will be able to interrupt completion at any point and resume later by creating their own access credentials. E-mails will be sent to participants whose survey has been incomplete for 1, 2 and 4 weeks, reminding them to complete it and offering them the opportunity to recover their access credentials if they have lost them.

For Study 3, the e-mail will contain the link to the form with information about the objectives of the study. Through this link, respondents can express their interest in participating, provide their contact details, and provide permission to be contacted by the research team to schedule the session. The sessions will take place in person in a space belonging to the university that hosts the project. The participants will have access to a computer on which the intervention platform will be made available. They will be instructed to guide their own use of the platform—with the aim of bringing this experience closer to its real use—while simultaneously receiving an opportunity to express their thoughts about it. If needed, the participants will be prompted to keep talking during the session. Video and audio recordings will be made, and written notes will be taken throughout the sessions for later analysis. To augment the information obtained during this task with a quantitative aspect, participants will also be asked to complete a self-report questionnaire at the end of the session.

In Study 4, an e-mail will contain a link to a website where interested respondents can find detailed information about the project and the intervention and through which they can complete a request to be contacted by the research team. This contact phone call will be used to explain the study and the intervention in more detail, answer questions, and verify eligibility. After eligibility is confirmed, the participants will be randomized into two groups: an experimental group, with immediate access to *AdoptMindful2Care@Web*, and a control group, with access to the intervention only at the end of the study. In both groups, participants will have to complete online self-report questionnaires, the link to which will be sent via e-mail, at three different times: T0 (preintervention), T1 (postintervention; for participants in the experimental group, the link will be sent 2 days after completing the last module of the intervention, and for the waiting group, 8 weeks after completing the T0 step), T2 (1-month follow-up), and T3 (2-month follow-up).

Informed consent will be obtained from all participants in the four studies after they have read the information provided about the research project, the inclusion criteria, the researcher’s duties, the participant’s rights, and the data protection policy used for data storage. In Studies 1, 2, and 4, consent will be obtained electronically by the participant selecting the option “Yes, I authorize”. In Study 3, written consent will be obtained.

The participants in the four studies will receive no financial compensation.

### 2.5. Sample Size and Statistical Analyses

In Study 1, since a universe of 137 adoption professionals is available, 57 will need to be recruited in order to achieve a confidence interval of 90%. Study 2 will require a minimum of 171 adoptive parents (95% CI, power of 0.90, medium-to-large effects, multivariate analyses; G*Power [53]). Statistical analyses will be conducted via the Statistical Package for the Social Sciences (IBM SPSS, version 27.0; IBM SPSS, Chicago, IL, USA). Descriptive statistics will be used to explore the adoptive parents’ sociodemographic, clinical, and child characteristics; e-health literacy; needs, preferences, and acceptance regarding *AdoptMindful2Care@Web*; and the adoption professionals’ characteristics and recommendations. Student’s t-test within subjects will be used to analyze the differences between the average number of identified advantages and disadvantages of *AdoptMindful2Care@Web* in both groups and between groups. This same test and chi-square tests will be used to compare other variables between both groups, specifically regarding the perception of usefulness and the acceptability of *AdoptMindful2Care@Web* (in the case of professionals, the report is based on what they perceive to be the opinion of adoptive parents). This comparison will also be conducted concerning the characteristics and functionalities of the platform (e.g., the most useful formats for presenting content) as well as the content itself (e.g., which topics are most relevant and pressing for these parents). These analyses will enable us to determine how the groups differ on these variables. Univariate and multivariate binary logistic regressions will be used to study the associations between independent variables (i.e., sociodemographic and child-related variables, mental health and parenting needs, and e-health literacy) and (1) the perceived utility of and (2) the availability to use *AdoptMindful2Care@Web*. The same will be conducted for adoption professionals to assess the associations between independent variables (i.e., sociodemographic and professional-related data) and the professionals’ (1) perceived utility of *AdoptMindful2Care@Web*, (2) perceptions about the parents’ availability to use *AdoptMindful2Care@Web*, (3) willingness to integrate *AdoptMindful2Care@Web* in their daily work, and (4) willingness to recommend *AdoptMindful2Care@Web* to the adoptive parents with whom they work.

For Study 3, a minimum of 15 individual Think-Aloud Protocol sessions will be held with adoptive parents. Qualitative data will be analyzed by using thematic analysis [54] via the NVivo14© software program. All video and audio recordings of the sessions will be transcribed. On the basis of these transcripts and notes taken during the sessions, usability issues will be identified and rated according to their severity: minor, medium, or critical. Bugs will also be flagged and later reported to web developers. All usability issues rated as medium or critical will be coded on the basis of their content and subsequently mapped according to two predefined categories: navigation or functionality. After this categorization, the categories will be cross-referenced with the general “usability heuristics for users interface design” [55]. These heuristics are often used by experts in the field of user-experience design to inform design decisions, since they offer a set of valuable guidelines developed over decades for designing systems, identifying usability problems and creating better user experiences. The quantitative data will be analyzed via SPSS. Descriptive statistics will be used to explore the perceptions of adoptive parents regarding the prototype version of *AdoptMindful2Care@Web*.

In Study 4, 60 participants (30 per condition; 95% CI, power of 0.99, medium effects, repeated measures ANOVA, and within–between interaction; G*Power [53]) will be needed in the final evaluation. Owing to the expected dropout rate of 60% in e-health interventions [56], we will recruit a total of 96 participants. Statistical analyses will be conducted via IBM SPSS. Descriptive statistics will be used to evaluate the feasibility and acceptability of *Adoptmindful2Care@Web*. Repeated measures ANOVAs/linear mixed models (timeXgroup interaction) will be used to analyze the modifications in participants’ outcomes at the four different assessment times.

In Studies 1, 2, and 4, sociodemographic, clinical, and/or child-related variables will be controlled for in all statistical analyses, in order to account for possible differences between adoptive parents.

### 2.6. Ethical Considerations

This project involves human participants and the collection/processing of personal data.

Ethical approval from the Faculty of Psychology and Education Sciences of the University of Coimbra has been obtained (CEDI/FPCEUC: 83/R_9 on 17 January 2024).

Anonymity/confidentiality requirements and the relevant legal contingencies will be respected at all stages and comply with international (Declaration of Helsinki; European Legislation) and national (Code of Ethics of the Ordem dos Psicólogos Portugueses [OPP]) ethical and deontological standards for human experimentation. The data protection guidelines of the University of Coimbra, which are governed by General Data Protection Regulation (GDPR), will also be strictly followed.

All data collection processes will begin after informed consent has been obtained from all participants. As adoption is not definitive in Portugal during the first months of children’s placement with prospective adopters, sample collection will only occur after the adoption has been finalized in court so as not to interfere with the adopters’ decision.

The participants will be informed about the voluntary nature of their participation and their right to refuse to participate, to access/change/delete/restrict the processing of their personal data, or to withdraw at any time without penalty and justification. They will also be assured that their data (including confidential personal data) will be kept anonymous, stored in secure databases (to which only the research team will have access), used only for research purposes, and kept only as long as necessary for the study. This information will be detailed on the home page of the online survey (Limesurvey^®^) hosted on the host institution’s website.

Ethical issues related to RCTs will also be considered. Participants who are not eligible for inclusion in the study groups and need appropriate psychological intervention will be counseled and referred for specialized follow-up. Guiding documents on clinical trial disclosure and data protection in clinical research from the National Commission for Ethics in Clinical Research will also be considered. The pilot RCT will be registered at clinicstrials.gov.

Given the development and study of web-based interventions, the OPP Guidelines for the Provision of Information and Communication Technology (ICT)-mediated Psychology Services will also be followed.

## 3. Expected Results

Data are planned to be collected up to May 2026.

In Study 1, detailed information will be collected regarding the recommendations of adoption professionals about the requirements *AdoptMindful2Care@Web* must satisfy to meet the needs of the adoptive parents (e.g., what the difficulties most frequently reported by such parents are and the types of help requested), their difficulties when providing support to this population (e.g., which topics should be addressed in the intervention), and the requirements *AdoptMindful2Care@Web* must satisfy to be integrated in Portuguese adoption services (e.g., if the intervention should be made available for the parents after the adoption report is issued, after the decree of the adoption by the court, or only after a parent help request).

Study 2 will provide detailed information about the literacy of adoptive parents regarding e-health tools (e.g., how useful such parents find the internet for health decision making, how accustomed they are to using the internet to locate health resources, and how knowledgeable they are about the resources available, how to find them and how to use them), the mental health and parenting needs that they expect to be addressed by *AdoptMindful2Care@Web* (e.g., which times they feel most challenged when playing the parental role), as well as their acceptance and preferences regarding the intervention (e.g., if they consider it potentially useful, if they would be available to participate in it, what are the advantages and disadvantages of this type of intervention format, which content types they prefer to have included in it [i.e., static or interactive content], and how many modules they consider adequate and the ideal length of each one).

Together, these results will inform the development of the content and technological features of *AdoptMindful2Care@Web* in a manner adjusted to the reality of its users and its application context. After the prototype version of the intervention is established, we will start collecting Study 3’s data regarding the usability of *AdoptMindful2Care@Web*. In this study, we will seek to obtain information about the users’ experience with *AdoptMindful2Care@Web* (e.g., what they find easy or difficult to understand and what they consider to be missing) and any issues that must be rectified. In turn, these results will guide the adaptations to be made to the intervention platform to create its pilot version.

Next, we will start Study 4, which will collect information about feasibility (e.g., how effectively participants are recruited and how well they stay engaged throughout the intervention, whether participants follow through with the prescribed contents and activities, and how practical the data collection methods are), acceptability (e.g., participants’ satisfaction, willingness to engage with the intervention, and feedback about its relevance and ease of use), and preliminary efficacy (i.e., improvements in parent and child outcomes at postintervention and/or at follow-up). With this comprehensive evaluation, we will be able to identify potential issues early on, facilitating any necessary adjustments in the intervention, as well as the optimization of the study design and protocol (e.g., randomization procedures, blinding, and the choice of outcome measures), so that later a larger RCT could be executed (with a subsequent study protocol).

## 4. Discussion

This paper describes a study protocol for the development and preliminary efficacy assessment of a web-based mindful parenting postadoption intervention (*AdoptMindful2Care@Web*). By employing a user-centered approach, this study protocol intends, first, to explore the recommendations regarding the intervention of professionals working in Portuguese adoption services (Study 1). Second, we aim to explore the e-health literacy of Portuguese adoptive parents, their mental health and parenting needs, and their preferences and acceptance (i.e., perceived utility and availability to use) regarding *AdoptMindful2Care@Web* (Study 2). Third, we aim to test the usability of the prototype version of *AdoptMindful2Care@Web* (Study 3). Finally, we intend to evaluate the intervention’s feasibility, acceptability, and preliminary efficacy (Study 4).

The development and provision of specialized psychological interventions for adoptive parents capable of addressing the specific challenges of this form of parenting is urgently needed worldwide. In Portugal, this urgency is underscored by resolution No. 373/2021 of the Assembly of the Republic, of December 29 [28], which recommends, at Point 10, “The implementation of integrated specialized responses to support families before, during and after the adoption processes, integrating formative, clinical, social and psycho-pedagogical responses”. However, this type of response is not yet available in the country, placing these parents—and, consequently, their children—at a disadvantage in regard to accessing specialized mental health care services. The evidence-based development of psychological interventions specifically designed to address adoptive parents’ needs and preferences and easy access would allow for important advancements in this matter.

Mindful parenting interventions seem to be an appropriate type of intervention for this population, one enabling an approach tailored to user needs [14,15]. Similarly, the use of e-mental health tools with this population appears to be a particularly promising means of overcoming the barriers posed by the structural organization of Portuguese adoption services with respect to providing face-to-face psychological interventions. E-health technologies applied in mental health fields represent an innovative form of intervention delivery, and recent studies have demonstrated that such technologies may be as effective as face-to-face interventions, with the additional benefit of reducing the need to physically attend interventions [57,58,59,60]. The application of the described intervention to families after child adoption will facilitate providing fast, practical, specialized, and effective support to these families.

Following the CeHRes Roadmap guidelines for user-centered development [25,26], our study protocol will ensure the evidence-based development and evaluation of a web-based mindful parenting postadoption intervention (*AdoptMindful2Care@Web*) adapted to adoptive parents—as prospective users—and adoption professionals—as mediators of intervention access. Both groups will be involved throughout the process of contextual inquiry, value specification, design, operationalization and summative evaluation of *AdoptMindful2Care@Web*. This approach could have pioneering implications for adoption research and practice, which we discuss next.

By involving Portuguese adoptive parents, Study 2 will contribute to closing a considerable gap in adoption research, which has overlooked the psychological impact of the challenges associated with the experience of adoptive parenthood on the well-being of these parents and their opinions on how they can be helped in the promotion of mental well-being [16]. Moreover, it will provide information about the literacy of these parents concerning e-health tools as well as their interest and willingness to use them, which will also be innovative. The information that will be contributed by this study concerning these specific issues could even be useful for decision making regarding other subsequent studies not necessarily related to this project, owing to the scope of its applicability. This information, together with the data obtained regarding specific preferences of adoptive parents concerning *AdoptMindful2Care@Web*, will serve to guide the development of the related web platform—regarding both the content it provides and the technological features it includes. Carrying out this study prior to the development of the intervention will increase the likelihood that *AdoptMindful2Care@Web* effectively meets the needs of these parents and serves its purpose [25]. However, to better ensure the suitability and effective future use of *AdoptMindful2Care@Web*, in addition to research solutions regarding its content and technological features, it will also be necessary to investigate the characteristics and strategies required for its effective implementation by Portuguese adoption services [26]. The results of Study 1 will address these topics since it elicits the views of adoption professionals working in the field, who are able to provide a more institutional view of the development and implementation of this intervention. In addition, the recommendations of these professionals about the difficulties reported daily by adoptive parents and their own difficulties in providing the support required by them will enrich the information obtained in Study 2. These data will contribute, once again, to the enhancement of knowledge in the field of the parenting and psychological functioning of Portuguese adoptive parents, ensuring the suitability of the intervention to the needs of the target population.

The results provided by Study 3 will also serve this objective, since they will give information on the aspects of the intervention that require modification to improve the user experience from the viewpoint of future users [20]. The same is true for Study 4, which will enable us to better understand the potential and weaknesses of the intervention in its actual context of use. This involvement of the key stakeholders of *AdoptMindful2Care@Web* is a strength of our study protocol, since it better ensures the suitability, usefulness, efficacy, and cost-effectiveness of the intervention among Portuguese adoptive parents.

By developing, and later making available for use, a web-based psychological intervention for this population that employs a user-centered approach by following the numerous steps proposed in this protocol, we will contribute to facilitating adoptive parents’ access to high-quality mental health services specifically tailored to them. This can subsequently contribute to reducing inequalities in access to adequate mental health care services and improving the well-being of families with adopted children, which, in turn, may prevent adoption dissolutions and ultimately increase the life opportunities of institutionalized children, who are vulnerable to chronic poverty and social exclusion [5].

Simultaneously, we also contribute to current research on the topic of e-health technologies for mental health care and to more effective management of resources in health care and adoption services, helping these services respond in a specialized and effective way to the psychological needs of adoptive parents.

Importantly, as far as we know, this will be the first study of this nature, namely, in the context of Portuguese adoption.

## 5. Conclusions and Limitations

In summary, this study protocol outlines a comprehensive approach to developing a mindful parenting web-based postadoption intervention tailored to the specific needs of Portuguese adoptive parents. By incorporating user-centered design principles, namely, engaging fathers, mothers, and professionals throughout the process, we aim to create a tool that not only enhances the mental well-being of adoptive families but also addresses the systemic challenges they face in accessing appropriate care.

Despite the promising approach outlined in this protocol, it is important to recognize several limitations. 

Firstly, since this is a web-based intervention, some limitations associated with technology use may arise, particularly regarding accessibility. Participants may have varying levels of digital literacy, which could hinder their ability to engage fully with the intervention. To address this issue, we will design a user-friendly and intuitive platform, ensuring ease of navigation and understanding for all users. In addition, telephone calls will be made to assist with any difficulties that may arise in this regard. Moreover, we understand that *AdoptMindful2Care@Web* may lack the personalization and therapeutic alliance of the face-to-face version, which could affect user satisfaction and overall efficacy. To mitigate this obstacle, we will opt for formats that in some way approximate the experience of real contact, namely, videos recorded by psychologists and audio recordings, enabling participants to engage with relatable content that fosters a sense of connection. We will also collaborate with a company specialized in behavioral science that will help us adapt the contents of the intervention, making them more attractive and better engaging for participants while motivating their commitment to the program. Moreover, we are aware that privacy concerns and data security present ethical challenges in digital health platforms, as users may be apprehensive about the confidentiality of their information. In this regard, we will employ robust encryption protocols, maintain transparent privacy policies, and ensure compliance with relevant data protection regulations, such as GDPR. 

Furthermore, the focus on Portuguese adoptive parents may restrict the applicability of the findings to different cultural settings. 

Lastly, given the preliminary nature of this study, further investigation, including a larger randomized controlled trial, will be essential to comprehensively evaluating the effectiveness and long-term benefits of *AdoptMindful2Care@Web*.

Despite these limitations, this protocol might be the starting point for the integration of e-mental health technologies in Portuguese adoption services.

## Figures and Tables

**Table 1:** An overview of the variables and anticipated outcomes of the studies and the measurement instruments.

Study	Variables/Outcomes	Measures	
**1** Adoption professionals	**Sociodemographic and Professional** **Information**	**Self-report datasheet, specifically developed by the research team, which will collect information regarding the following:** (1) Sociodemographic data: 3 questions with multiple-choice exclusive answer; e.g., sex, age, and educational level; (2) Professional data: 9 questions with multiple-choice exclusive answer; e.g., position currently held and geographical area of direct activity.	
	**Recommendations regarding *AdoptMindful2Care@Web***	**Self-report datasheet, specifically developed by the research team, which will collect information regarding the characteristics required from *AdoptMindful2Care@Web* to meet the following:** (1) The needs usually expressed by adoptive parents to adoption teams: 4 questions with multiple-choice exclusive answer and multiple-choice questions with several possible answers; e.g., parenting difficulties most commonly reported by adoptive parents to professionals and the moment in which the difficulties are reported to professionals. (2) The adoption professionals’ own difficulties in providing support: 11 questions with multiple-choice exclusive answer, multiple-choice questions with several possible answers, 5-point Likert scale and yes–no answers; e.g., to what extent they think they have enough knowledge and enough time to respond to parents’ requests and level of comfort in providing support to parents in specific areas of difficulty. (3) The ethical and institutional requirements for the integration of the intervention in Portuguese adoption services: 15 questions with multiple-choice exclusive answer, multiple-choice questions with several possible answers, 5-point Likert scale, yes–no answers, and open answers.	
**2** Adoptive parents	**Sociodemographic information**	**Self-report datasheet, specifically developed by the research team:** 15 questions with multiple-choice exclusive answer and open answers; e.g., sex, age, marital status, educational level, professional and socioeconomic status, and number of household members.	
	**Child-related** **information**	**Sociodemographic data**	**Self-report datasheet, specifically developed by the research team:** 4 questions with multiple-choice exclusive answer and open answers; e.g., number of adopted children, age of children, and time since the adoption was decreed by the court.
		**Socioemotional development**	**Children < 2 years old** **Baby Pediatric Symptom Checklist** (BPSC [30]; PV: [31]): hetero-report measure; 12 items divided into 3 sub-scales; Likert scale (ranging from 0 = no to 2 = very); a total score of 3 or more points in any of the sub-scales indicates that the child is “at risk”.
			**Children 2–5 years old** **Preschool Pediatric Symptoms Checklist** (PPSC [32]; PV: [33]): hetero-report measure; 18 items divided into 4 dimensions; Likert scale (ranging from 0 = no to 2 = very); scores of 9 or more indicate that the child is “at risk”.
			**Children 6–17 years old** **Pediatric Symptom Checklist** (PSC17 [34]): hetero-report measure; 17 items divided into 3 sub-scales; Likert scale (ranging from 0 = never to 2 = frequently); higher scores indicate a greater risk of problems in psychosocial functioning.
			**Children 2–4 and 4–17 years old** **Strengths and Difficulties Questionnaire** (SDQ [35]): hetero-report measure; 25 items divided into 5 sub-scales; Likert scale (ranging from 0 = not true to 2 = certainly true); higher total difficulty scores indicate a greater risk of behavioral and emotional difficulties.
	**Mental health and parenting needs**	**Positive mental health**	**Mental Health Continuum–Short Form** (MHC–SF [36]; PV: [37]): self-report measure; 14 items divided into 3 dimensions; Likert scale (ranging from 0 = never to 5 = every day); higher scores indicate higher levels of positive mental health.
		**Parenting stress**	**Parenting Stress Index** (PSI-SF [38]; Portuguese Version (PV): [39,40]): self-report measure; 35 items divided into 3 sub-scales; Likert scale (ranging from 1 = strongly disagree to 5 = strongly agree); higher scores indicate higher levels of parenting stress.
		**Mindful parenting**	**Parents with children < 2 years old** **Interpersonal Mindfulness in Parenting Scale–Baby** (IM-P-B [41]; PV: [42]): self-report measure; 30 items divided into 5 dimensions; Likert scale (ranging from 1 = never to 5 = always); higher scores indicate higher levels of mindful parenting.
			**Parents with children > 2 years old** **Interpersonal Mindfulness in Parenting Scale** [IM-P [41]; PV: [43]): self-report measure; 31 items divided into 5 dimensions; Likert scale (ranging from 1 = never to 5 = always); higher scores indicate higher levels of mindful parenting.
	**E-health literacy**	**Portuguese version of the E-Health Literacy Scale** [44]: Self-report measure; 8 items; Likert scale (ranging from 1 = strongly disagree to 5 = strongly agree). Higher scores indicate higher levels of e-health literacy.	
	**Preferences and acceptability of an e-health intervention**	**Self-report datasheet, specifically developed by the research team, which will collect the information regarding the characteristics required of *AdoptMindful2Care@Web* to meet the adoptive parents’ acceptability and preferences regarding the intervention:** 22 questions with multiple-choice exclusive answer, multiple-choice questions with several possible answers, 5-point Likert scale, and yes-no answers; e.g., whether they would find it useful, whether they would be interested in participating, and preferences for content formats.	
**3** Adoptive parents	**Usability of *AdoptMindful2Care@Web***	**The Think-Aloud Protocol** will be used to examine how adoptive parents experience *AdoptMindful2Care@Web* in the context of its actual use. **A self-report datasheet**, specifically developed by the research team, will be used to collect quantitative data regarding the prototype version of *AdoptMindful2Care@Web* (e.g., how appealing and intuitive adoptive parents find the platform).	
**4** Adoptive parents	**Acceptability of *AdoptMindful2Care@Web***	**Self-report datasheet, specifically developed by the project team, which will collect information regarding the satisfaction with the contents and characteristics of the intervention and the opinions regarding the intervention’s quality and relevance:** 25 questions; Likert scale and multiple-choice exclusive answer; e.g., whether taking part in the intervention was useful, whether it helped to improve their relationship with their child, whether they would recommend the intervention to family or friends, whether they consider the technological resources provided by the platform to be adequate, the importance attributed to the different contents covered, and the usefulness attributed to the different technological tools provided.	
	**Feasibility of *AdoptMindful2Care@Web***	**Adherence rate, dropout rate, and web system data** (e.g., number of logins, the average time of access to the program at each login, number of answers to the exercises, and number of times the contents were repeated).	
	**Preliminary efficacy of *AdoptMindful2Care@Web* (evaluated at T0, T1, T2, and T3)**	Parenting stress *	**Parenting Stress Index** (PSI-SF [38]; Portuguese Version (PV): [39,40]): described above.
		Positive mental health *	**Mental Health Continuum–Short Form** (MHC–SF [36]; PV: [37]): described above.
		Mindful parenting **	**Parents with children < 2 years old** **Interpersonal Mindfulness in Parenting Scale–Baby** (IM-P-B [41]; PV: [42]): described above.
			**Parents with children > 2 years old** **Interpersonal Mindfulness in Parenting Scale** [IM-P [41]; PV: [43]): described above.
		Parental reflective functioning **	**Parental Reflective Functioning Questionnaire** (PRFQ [45]; PV: [46]): self-report measure; 18 items divided into 3 sub-scales; Likert scale (ranging from 1 = strongly disagree to 5 = strongly agree). Higher scores on the Pre-Mentalizing Modes sub-scale indicate more maladaptive reflective functioning. Higher scores on the Certainty and Curiosity sub-scales indicate more adaptive parental reflective functioning.
		Depression and anxiety **	**Hospital Anxiety and Depression Scale** (HADS [47]; PV: [48]): self-report measure; 12 items divided into 2 sub-scales; Likert scale (ranging from 0 = not at all/just occasionally to 3 = most of the time/most of the time); higher scores indicate higher levels of symptomatology.
		Self-compassion **	**Self-Compassion Scale–Short Form** (SCS-SF [49]; PV: [50]): self-report measure; 12 items divided into 6 sub-scales; Likert scale (ranging from 1 = almost never 5 = almost always); higher scores indicate higher levels of self-compassion.
		Mindfulness **	**Mindful Attention and Awareness Scale** (MAAS [51]; PV: [52]): self-report measure; 15 items; Likert-scale (ranging from 1 = almost always to 6 = almost never); higher scores indicate higher levels of mindfulness.
		Child’s behavioral and emotional difficulties **	**Children < 2 years old** **Baby Pediatric Symptom Checklist** (BPSC [44]; PV: [45]): described above.
			**Children 2–5 years old** **Preschool Pediatric Symptoms Checklist** (PPSC [46]; PV: [47]): described above.
			**Children 6–17 years old** **Pediatric Symptom Checklist** (PSC17 [48]): described above.
			**Children 2–4 and 4–17 years old** **Strengths and Difficulties Questionnaire** (SDQ [49]): described above.

Note: T0 = baseline assessment; T1 = postintervention assessment; T2 = 1-month-follow-up assessment; T3 = 2-month-follow-up assessment. * Primary outcomes. ** Secondary outcomes.

## Data Availability

Data sharing is not applicable to this article.
